# A new strategy for volumetric-modulated arc therapy planning using AutoPlanning based multicriteria optimization for nasopharyngeal carcinoma

**DOI:** 10.1186/s13014-018-1042-x

**Published:** 2018-05-16

**Authors:** Juanqi Wang, Zhi Chen, Weiwei Li, Wei Qian, Xiaosheng Wang, Weigang Hu

**Affiliations:** 1 Department of Radiation Oncology, Fudan University Shanghai Cancer Center, Shanghai, China; 2 Department of Oncology, Shanghai Medical College, Fudan University, Shanghai, China

**Keywords:** Volumetric-modulated arc therapy, Multicriteria optimization, Automated planning optimization, Patient-specific tradeoff, Nasopharyngeal carcinoma

## Abstract

**Background:**

A new strategy for making the appropriate choice of the representative optimization parameters in planning processes and accurate selection criteria during Pareto surface navigation for general multicriteria optimization (MCO) was recommended in the study. The purpose was to combine both benefits of AutoPlanning optimization and MCO (APMCO) for achieving an individual volumetric-modulated arc therapy (VMAT) plan according to the clinically achieved patient-specific tradeoff among conflicting priorities. The preclinical investigation of this optimization approach for nasopharyngeal carcinoma (NPC) radiotherapy was performed and compared to general MCO VMAT.

**Methods:**

A total of 60 NPC patients with various stages were enrolled in this study. General MCO and APMCO plans were generated for each patient on the treatment planning system. The differences between two planning schemes were evaluated and compared.

**Results:**

All plans were capable of achieving the prescription requirement. The planning target volume coverage and conformation number were remarkably similar between general MCO and APMCO plans. There were no significant differences in most of organs at risk (OARs) sparing. However, in APMCO plans, relatively remarkable decreases were observed in the mean dose (D_mean_) to the glottic larynx and pharyngeal constrictor muscles. The reductions of average D_mean_ to the two OARs were 10.5% (*p* < 0.0001) and 8.4% (*p* < 0.0001), respectively. APMCO technique was found to increase the planning time for an average of approximately 5 h and did not lead to a significant increase of monitor units compared to general MCO.

**Conclusions:**

The potential of the APMCO strategy is best realized with a clinical implementation that exploits individual generation of Pareto surface representations without manual interaction. It also assists physicians to ensure navigation in a more efficient and straightforward manner.

## Background

Volumetric-modulated arc therapy (VMAT) has experienced a rapid and widespread clinical application due to the similar or superior plan quality to the fixed field intensity modulated radiation therapy [1–3]. However, a challenging problem for VMAT is that the optimal treatment plan generally depends on the planner’s devoted time and experience [4] to translate the clinical goals into optimization parameters accounting for the relation among three competing priorities: planning target volume (PTV) coverage, PTV dose homogeneity, and sparing of the adjacent organs at risk (OARs). More concern seems to be attracted especially for the complicated head and neck cancer, such as nasopharyngeal carcinoma (NPC) where multiple PTV dose levels are defined close to OARs. Recently, multicriteria optimization (MCO) techniques have been introduced into VMAT planning [5–9]. The rationale of multicriteria VMAT optimization is to first efficiently explore a Pareto-optimal tradeoff between conflicting priorities in the fluence domain, and second fine-tune the tradeoff with respect to deliverable linear accelerator settings and final dose calculated.

In principle, MCO enables planners to determine a best-possible compromise using an ideal Pareto surface. Even with MCO, there remains a manual selection bias owing to variations in the management of clinical tradeoffs. In general, the choice of optimization parameters in planning processes [10–13] and selection criteria during Pareto surface navigation [14, 15] may yield different tradeoffs in MCO. Different solutions may produce tradeoffs among different choices. Without the patient-specific choices, MCO planning also needs a manual trial-and-error process similar to traditional inverse planning. In many situations, subjectivity in which tradeoffs are made results in suboptimal plans being delivered in the clinical routine practice, resulting in worse patient outcomes. The great concern and difficulty in MCO planning is how to determine the representative optimization parameters and accurate selection criteria for the optimal Pareto plan. Because of the difficulty in lacking the accurate tradeoff information, there are no guarantees on deriving the optimality of solutions after the numerous continuing searches. Although this problem has been addressed by a number of research groups over the past 10 years [16–20], there is still no good solution for automatically finding the optimal choice.

Recently, AutoPlanning (AP) approach has been incorporated in clinical processes to improve plan quality and efficiency [21, 22]. The concept is largely to capture the steps that an experienced planner would take and then simulate them for a new patient. AP employs an iterative algorithm approach to reach and potentially surpass clinical goals that planners defined. Individual optimization goals, constraints and weights are automatically added and adjusted during AP. The optimizer will run multiple times with adjustments being made during and between optimization runs. Additionally, AP adjusts the priority of clinical goals based on the probability of being achieved. Although AP appears to have difficulty in determining a Pareto-optimal compromise, it would be a fully automated planning process to efficiently explore a patient-specific tradeoff.

The quality of plans generated by the MCO approach was evaluated by comparison with benchmark plans generated by the conventional manual approach [9, 15, 20]. MCO has proven to be an efficient approach, both in terms of dosimetric quality and planning efficiency. A notable difference to previous work is that we focused on recommending a new strategy for making the appropriate choice of the representative optimization parameters in planning processes and accurate selection criteria during Pareto surface navigation for general MCO. The purpose of this study was to combine both benefits of AP optimization and MCO (APMCO) for achieving an individual VMAT plan according to the clinically achieved patient-specific tradeoff among conflicting priorities. The preclinical investigation of this optimization approach for NPC radiotherapy was performed and compared to general MCO VMAT.

## Methods

### Patient selection and contouring

Considering a heterogeneous patient collective, a total of 60 NPC patients treated with VMAT between January 2016 and September 2017 were consecutively enrolled in this study. All patients were immobilized in the supine position with a head, neck and shoulder thermoplastic mask. The patients’ characteristics are presented in Table 1. None of the patients had received prior radiotherapy, and all were free of distant metastases. Computed tomography with a 3 mm slice thickness of the head and neck region was obtained for each patient and imported to the treatment planning system (TPS). According to the Radiation Therapy Oncology Group (RTOG) protocols 0225 and 0615, the same attending physician delineated the target area and OARs for all patients. The target area included three clinical target volumes (CTVs): CTV_70_, defined as 70 Gy radiation to the nasopharyngeal gross tumor and lymphadenopathy; CTV_59.4_, defined as 59.4 Gy radiation to the high-risk lymphatic regions; and CTV_54_, defined as 54 Gy radiation to the low-risk regions. The PTV provided a 3 mm margin around the CTV to compensate for the variability of treatment set up and internal organ motion, except for the situation that the CTV was adjacent to the brain stem, where the margin could be as small as 1 mm. The OARs included the brain stem, spinal cord, parotid glands, optic nerves, chiasm, temporal lobes, oral cavity, lenses, glottic larynx, submandibular glands and pharyngeal constrictor muscles.

**Table 1:** Patient characteristics (*N* = 60)

Variables	Number
Age, median years (range)	46(25-72)
Gender	
Male	38(63.3%)
Female	22(36.7%)
AJCC stage	
I	14(23.3%)
II	15(25%)
III	16(26.7%)
IVA-B	15(25%)

Abbreviation: *AJCC* American Joint of Cancer Committee in 2010

### Dose prescription and treatment planning

The commonly used simultaneously integrated boost technique was adopted at our institution. The dose prescribed to PTV_70_ was 2.12 Gy per fraction, to PTV_59.4_ was 1.8 Gy per fraction and PTV_54_ was 1.64 Gy per fraction in 33 fractions, respectively. We adopted the Varian Trilogy linear accelerator (Varian Medical Systems, Palo Alto, CA, USA) to compare general MCO VMAT to APMCO VMAT, and 6MV photon beams were applied to all plans. The treatment goals were that 100% of the prescribed dose would cover 95% of the PTV received three dose levels, and the maximum dose would not exceed 110%. The dose-volume constraints to critical OARs from the RTOG protocols 0225 and 0615 are described in Table 2. According to the International Commission on Radiation Units and Measurements report 83, the near minimum dose (D_98%_) was the dose to 98% of the structure, the near maximum dose (D_2%_) was defined to be the dose to 2% of the structure and the mean dose (D_mean_) was the mean dose to the structure. Regarding the OARs, D_2%_ to the brain stem, the spinal cord, the optic nerves and the chiasm was set as 54 Gy, 45 Gy, 54 Gy, and 54 Gy, respectively. In addition, at least one of the parotid glands should receive a D_mean_ of no more than 26 Gy, or at least 50% of one gland should receive < 30 Gy.

**Table 2:** The dose-volume constraints to critical OARs used in VMAT optimization

OAR	Dose constraint
Brain stem	D_2%_ < 54 Gy
Spinal cord	D_2%_ < 45 Gy
Parotid glands	D_mean_ < 26 Gy or at least 50% of one side will receive <30Gy
Optic nerves, Chiasm	D_2%_ < 54 Gy
Temporal lobes	D_2%_ < 60 Gy
Oral cavity (excluding PTV’s)	D_mean_ < 40 Gy
Lens	D_2%_ < 10 Gy
Glottic larynx	D_mean_ < 45 Gy
Submandibular glands	D_mean_ < 39 Gy
Pharyngeal constrictor muscles	D_mean_ < 50 Gy

Abbreviations: *D*_*2%*_ dose to 2% of the structure, *D*_*mean*_ mean dose

The VMAT plans, described earlier [23], were generated using two complete arcs plus one partial arc (moving from gantry angle of 240° to 120° in the clockwise direction). The collimator angle was set at 20° for all plans to minimize the cumulative effects of interleaf transmission and the tongue-and-groove effect. The 3° was set as a spacing unit in this study for the compromise between the number of optimizable multileaf collimator leaf positions and calculation time. The same Trilogy linear accelerator data was commissioned in the Pinnacle TPS (Version 9.10, Philips Radiation Oncology Systems, Fitchburg, WI) and RayStation TPS (Version 3.0, RaySearch Laboratories AB, Stockholm, Sweden). The dose differences between the two systems were verified within 1.5% during the commission process [24].

### Description of AP

A new optimizer, AP, was introduced in Pinnacle TPS. The AP module simplifies the planning process through the use of templates (derived regions of interest, placement of points of interest, prescriptions, beam geometries, settings, and optimization options and prioritized optimization goals), and automatic optimization tuning algorithm (called the AP engine). A single selection creates a new plan based on the templates and runs the AP engine. The AP engine maps the prioritized optimization goals defined in the templates to optimization objectives. Multiple optimization loops are performed that iteratively adjust the optimization parameters to meet the goals and further drive down OAR sparing with minimal compromise to the PTV coverage. It is achieved by using parameters specific to driving down OAR dose to the point that it significantly affects PTV coverage and separate parameters to achieve the planning goals. PTV conformity and uniformity is automatically controlled by particular system-generated structures. It is similar to the process defined in the study by Xhaferllari et al. [21]. OAR dose is controlled by normal tissue structures. All the optimization parameters (dose and weight) are adjusted using a proprietary approach. In brief, the AP software is analogous to experienced planners drawing new structures and adjusting parameters to make inverse plans more clinically desirable.

### Description of MCO and clinical workflow

**Fig. 1:**
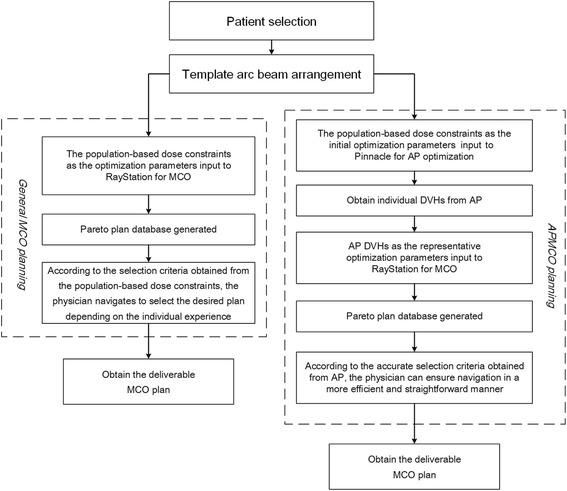
The workflow of general MCO VMAT and APMCO VMAT treatment

MCO has been commercialized in RayStation TPS. The whole workflow of general MCO VMAT and APMCO VMAT treatment is schematically presented in Fig. 1. There are mainly three stages in general MCO planning process: in the first stage, the TPS generates Pareto-optimal plans according to the typical dose-volume constraints input as the optimization parameters for the multicriterial planning problem and stores them in a database. In the planning process, no sufficient quantitative judgments are used to reduce adverse effects that can jeopardize survival advantages other than a set of dose-volume constraints. The most common constraints have typically been determined using the population-based constraints such as RTOG protocol recommendations. In the second stage, the physician interactively explores the Pareto surface and decides for the best-possible compromise for the patient, depending on the individual experience. In the last stage, the deliverable plan is finalized by multileaf collimator sequencing and final dose calculation. The Pareto surface approximation is calculated by optimizing various weighted sums of the objective cost functions. For N functions at least N + 1 plans in the Pareto database are calculated [10], and the maximum plan number is given by the TPS with 4 N (default usage for the remarkable performance in this study).

The overall workflow of APMCO VMAT is quite similar in general. The major differences were the optimization parameters and selection criteria. For general MCO, the optimization parameters and selection criteria were directly from the population-based dose constraints. For APMCO, the population-based dose constraints as the initial optimization parameters were input to Pinnacle for AP optimization. After AP progress, the individual AP dose-volume histograms (DVHs) as the representative optimization parameters were used as the input for MCO in RayStation. During final plan selection in the navigation step, APMCO can make the physician who is confronted with the full range of alternative choices ensure navigation in a more efficient and straightforward manner according to the accurate selection criteria obtained from AP instead of the individual experience.

### Dose comparisons

Plan comparisons were performed between general MCO and APMCO plans. In order to minimize the influence of the inter-operator variation, the same planner and physician adopting the same plan criteria were engaged in this study. DVHs were calculated for all plans. For PTVs, D_98%_ and D_2%_were compared. The conformity of the PTV dose was evaluated by the conformation number (CN) according to the following eq. (1):

1 $$ \mathrm{CN}=\frac{{{\mathrm{PTV}}^2}_{\mathrm{ref}}}{{\mathrm{V}}_{\mathrm{PTV}}\times {\mathrm{V}}_{\mathrm{ref}}}, $$

where PTV_ref_ represented the volume receiving the prescription dose in PTV, V_PTV_ stood for the volume of the PTV, and V_ref_ was the volume that received the prescribed dose. For OARs, D_2%_ was applied to evaluate the dose to serial organs, such as the brain stem, spinal cord, optic nerves, chiasm and temporal lobes, and D_mean_ to parallel organs, such as the parotid glands, oral cavity (excluding PTV’s), glottic larynx, submandibular glands and pharyngeal constrictor muscles. For parotid glands, the percent volume of each parotid that received 30 Gy (V_30_) was also evaluated and compared. The plan difference was facilitated by defining the relative OAR dose deduction (δ) according to the following eq. (2):

2 $$ \updelta =\frac{{\mathrm{P}}_{GeneralMCO}\hbox{-} {\mathrm{P}}_{\mathrm{APMCO}}}{{\mathrm{P}}_{GeneralMCO}}\times 100, $$

where P refers to the dose (Gy).

The number of monitor units (MUs) for all plans and planning time including the time spent for optimization and dose calculation until the final plan was produced were also reported.

### Statistical analysis

Statistical analysis was performed to compare the dosimetric differences between general MCO and APMCO plans. The normal distribution of variables was firstly checked with the Kolmogorov-Smirnov test. Paired t-tests were used to compare the different parameters. A *p*-value < 0.05 was considered statistically significant. All statistical analyses were performed with IBM-SPSS statistics, version 19 (SPSS Inc., Chicago, IL).

## Results

### PTV coverage and CN

**Fig. 2:**
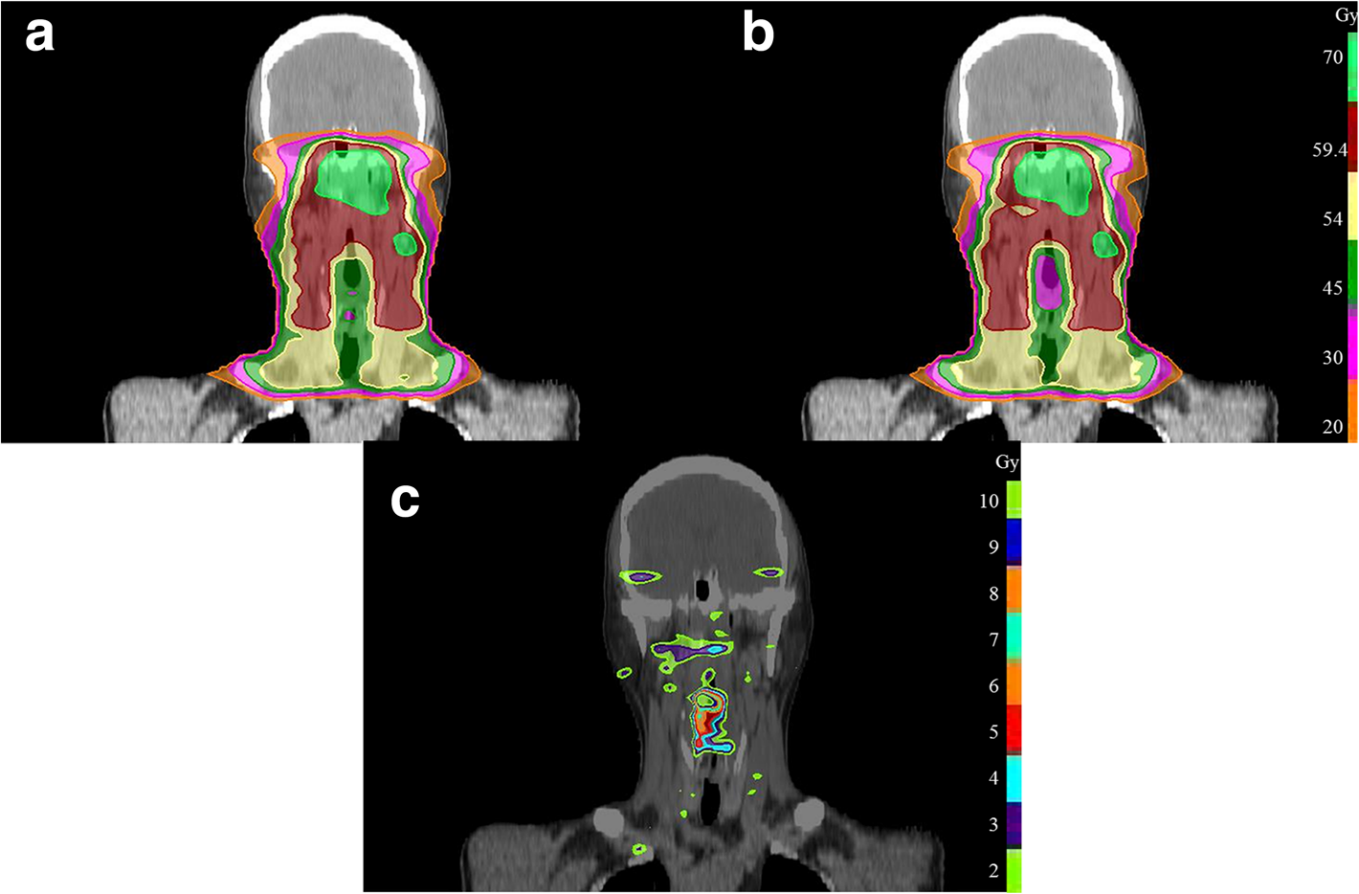
The coronal dose distributions for **a** general MCO VMAT and **b** APMCO VMAT are shown for one representative patient. **c** displays the dose difference between (**a**) and (**b**)

The dose distributions for one representative NPC patient on coronal planes of general MCO and APMCO plans are shown in Fig. 2a–b, respectively. Table 3 presents the detailed statistical analysis of PTVs, which are averaged over 60 patients. All plans were capable of achieving the prescription requirement. There were almost no statistically significant differences between general MCO and APMCO plans in terms of PTV_70_, PTV_59.4_, PTV_54_ coverage and CN. It clearly showed that the PTV dose distributions were essentially equivalent between the two plan types.

**Table 3:** Summary of the dosimetric parameters from the investigated techniques of PTVs, OARs and MUs

Item	Parameter	General MCO-VMAT	APMCO-VMAT	*p* value
PTV_70_	D_98%_ (Gy)	69.5 ± 0.3	69.4 ± 0.5	0.66
	D_2%_ (Gy)	74.7 ± 0.9	74.9 ± 0.9	0.11
	CN	0.6 ± 0.1	0.6 ± 0.1	0.50
PTV_59.4_	D_98%_ (Gy)	58.1 ± 0.7	58.3 ± 0.7	0.09
	D_2%_ (Gy)	74.0 ± 0.6	74.1 ± 0.6	0.29
	CN	0.5 ± 0.0	0.5 ± 0.1	0.89
PTV_54_	D_98%_ (Gy)	53.2 ± 0.9	53.0 ± 0.8	0.31
	D_2%_ (Gy)	60.0 ± 0.9	60.1 ± 0.9	0.64
	CN	0.2 ± 0.0	0.2 ± 0.0	0.15
Brainstem	D_2%_ (Gy)	51.1 ± 2.1	50.9 ± 2.3	0.59
Spincal cord	D_2%_ (Gy)	42.1 ± 1.8	41.8 ± 1.9	0.31
Left optic nerve	D_2%_ (Gy)	46.2 ± 6.8	46.3 ± 7.0	0.74
Right optic nerve	D_2%_ (Gy)	45.7 ± 7.4	45.2 ± 7.5	0.13
Chiasm	D_2%_ (Gy)	41.8 ± 8.7	41.5 ± 9.4	0.67
Left len	D_2%_ (Gy)	6.1 ± 1.0	6.1 ± 1.1	0.77
Right len	D_2%_ (Gy)	6.1 ± 1.0	6.1 ± 1.1	0.93
Left parotid gland	V_30_ (%)	42.1 ± 5.6	42.7 ± 2.5	0.17
	D_mean_ (Gy)	31.8 ± 1.7	31.2 ± 4.0	0.41
Right parotid gland	V_30_ (%)	42.5 ± 2.7	42.7 ± 2.9	0.24
	D_mean_ (Gy)	31.2 ± 3.9	31.9 ± 1.7	0.16
Left temporal lobe	D_2%_ (Gy)	63.2 ± 6.3	63.5 ± 6.7	0.34
Right temporal lobe	D_2%_ (Gy)	64.5 ± 5.7	64.9 ± 5.7	0.36
Oral cavity (excluding PTV’s)	D_mean_ (Gy)	39.9 ± 2.7	39.7 ± 3.1	0.35
Glottic larynx	D_mean_ (Gy)	50.9 ± 3.3	45.6 ± 3.3	< 0.0001
Submandibular glands	D_mean_ (Gy)	55.1 ± 1.8	54.9 ± 2.1	0.55
Pharyngeal constrictor muscles	D_mean_ (Gy)	53.8 ± 1.4	49.3 ± 1.4	< 0.0001
MU		505.6 ± 40.5	510.5 ± 34.3	0.09

Abbreviations: *PTV* planning target volume, *D*_*98%*_ dose to 98% of the structure, *D*_*2%*_ dose to 2% of the structure, *CN* conformation number, *V*_*30*_ volume (%) of receiving 30 Gy, *D*_*mean*_ mean dose, *MU* monitor unit

Data are shown as mean values with one standard deviation

### OARs sparing

**Fig. 3:**
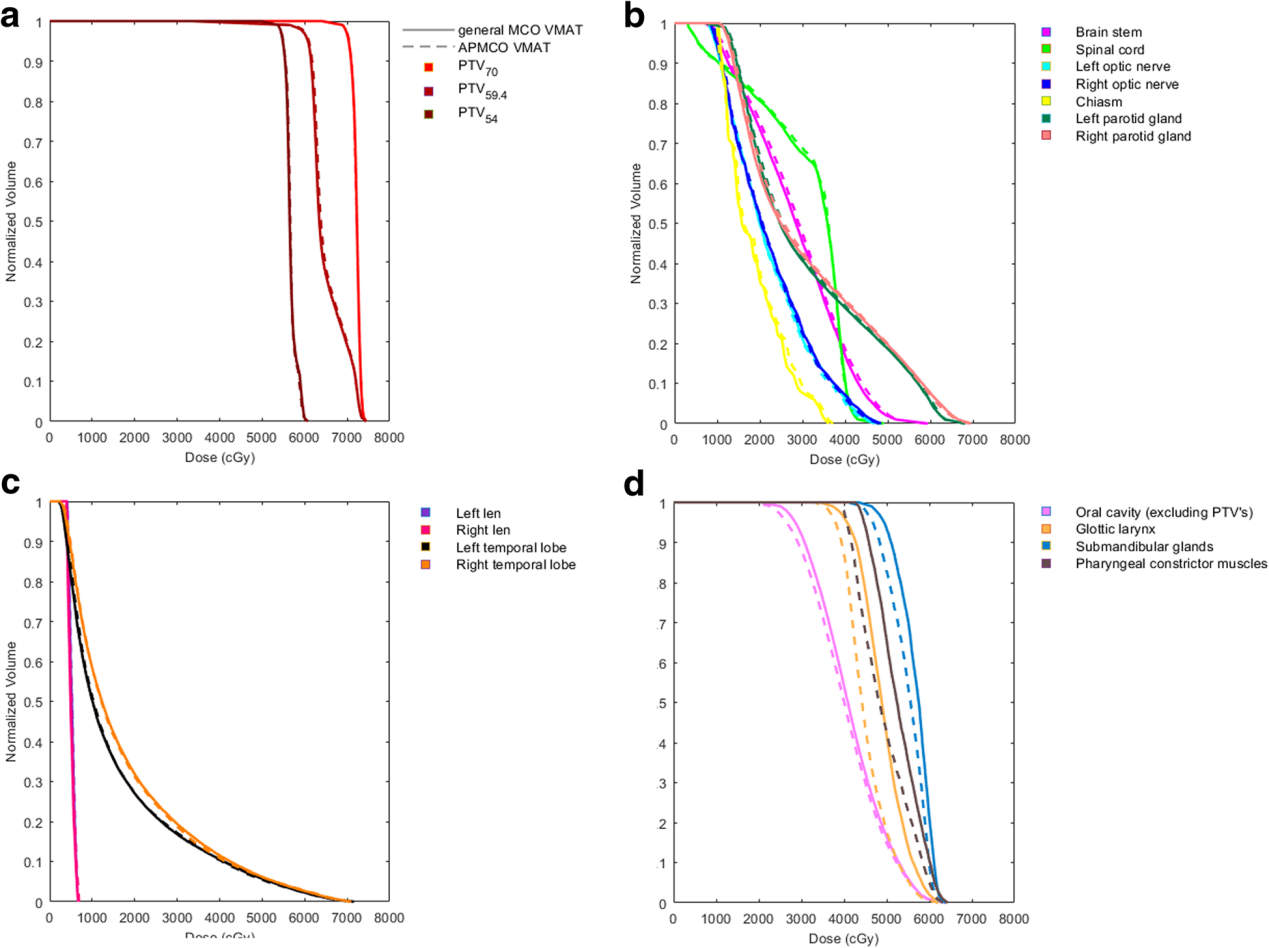
The mean DVHs for PTVs and OARs comparing general MCO (solid lines) to APMCO (dotted lines). **a** PTV_70_, PTV_59.4_ and PTV_54_ **b** Brain stem, spinal cord, optic nerves, chiasm and parotid glands **c** Lens and temporal lobes **d** Oral cavity (excluding PTV’s), glottic larynx, submandibular glands and pharyngeal constrictor muscles

**Fig. 4:**
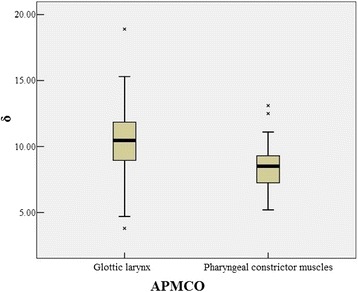
The box plot depicting the effect of APMCO implementation on glottic larynx and pharyngeal constrictor muscles dose. δ means the relative OAR mean dose deduction. The center line inside box is the median value. The box represents the interquartile range (IQR) from 25% quartile to 75% quartile. The cross indicates the outlier which is defined as the points more than 1.5 times IQR away from the box edge. The solid lines connect the box with the most extreme values which are not outlier

Figure 2c displays the dose differences between Fig. 2a, and Fig. 2b. Fig. 3 shows the mean DVHs of all structures. The distributions of all DVH metrics were sufficiently similar to normal distributions. The major dose differences occurred in the glottic larynx and pharyngeal constrictor muscles. Table 3 also summarizes the dosimetric parameters from the investigated techniques of OARs. There were no significant differences in most of OARs sparing. However, in APMCO plans, relatively remarkable decreases were observed in D_mean_ to the glottic larynx and pharyngeal constrictor muscles. The reductions of average D_mean_ to the two OARs were 10.5% (*p* < 0.0001) and 8.4% (*p* < 0.0001), respectively. Figure 4 illustrates the effect of APMCO implementation on glottic larynx and pharyngeal constrictor muscles dose. The results suggested that APMCO technique could more effectively protect the glottic larynx and pharyngeal constrictor muscles.

### Planning efficiency and MUs

Multiple PTVs and OARs that needed to be balanced in NPC treatment resulted in a time-consuming process. An average of approximately 5 h was required to create each MCO plan. APMCO technique significantly increased the planning time. Additional average of approximately 5 h was required to create each AP plan. The mean number of MUs for APMCO plans was 505.6 compared to 510.5 for general MCO ones (*p* > 0.05) (Table 3). The use of APMCO did not lead to a significant increase of MUs.

## Discussion

In this study, a new optimization approach was developed for VMAT planning. We demonstrated the feasibility of implementing APMCO to assist VMAT. NPC chosen for the analysis represented an ideal platform for this study. Besides its relatively high incidence in Southeast Asia and China, there are multiple PTVs and a great deal of individual radiosensitive OARs that need to be balanced, testing APMCO performance in a demanding clinical scenario. Our results showed that APMCO plans maintained similar PTV coverage and CN, and similar or superior OARs sparing to general MCO ones. As more agents are tested in combination with radiation in attempts to improve the efficacy of chemoradiation, it is highly desirable to explore new strategies for NPC patients that will decrease radiation-specific adverse effects. The quantitative analysis of normal tissue effects in the clinic project [25] reviewed several dose-response studies for laryngeal edema, vocal dysfunction and dysphagia. It found that the probability of these adverse effects increased significantly with the dose to the glottic larynx and pharyngeal constrictor muscles. In our study, the relevantly improved sparing of the two OARs was achieved after implementing APMCO without compromising PTV coverage, homogeneity, and sparing of the rest of OARs. It was suggested that the dose reduction in these two tissues did not result from the redistribution of the dose to PTVs and other OARs, but rather from the utilization of APMCO. Furthermore, additional reduction of potential treatment adverse effects may offer the opportunity to effectively improve the quality of life.

In the planning process, the idea underlying the MCO formulation is that each anatomical structure is assigned one or several parameters. Therefore, even with MCO the optimization results depend heavily on the optimization parameters. This is an open question as to which sets of choices best achieve the appropriate balance of dose distribution. Craft et al. [10] demonstrated that the resulting generation of the Pareto surface was sensitive to the used parameters particularly for some parallel OARs, where D_2%_ and D_mean_ were not correlated. Therefore, a key challenge in MCO is to define the patient-specific parameters that will directly affect the Pareto surface generation. Various solutions have been proposed to assist planners to efficiently balance clinical tradeoffs and decide the clinically achievable plans, such as knowledge-based planning (KBP) [26–37] and AP [21, 22]. KBP has proven to be an efficient tool for significantly improving planning efficiency and stability. More importantly, adoption of KBP led to varying degrees of improved OARs sparing for head and neck cancer patients, such as parotid glands, oral cavity, glottic larynx and cochlea [32, 33]. A key pre-requisite of KBP is to compile a sufficiently large number of high quality plans to build a DVH estimation model. One disadvantage is the range of patient geometries in the model, which still may not represent the full diversity of NPC cases due to individual differences. The estimations and statements about the accuracy of the model may be compromised to a certain extent when applying the model to those patients whose geometry falls outside the range of the constituent plans in the model. Another disadvantage is that the performance of KBP mainly depends on the quality of model. Suboptimal plans in the model may degrade results with the KBP approach. Furthermore, the predicted goals are directly applied to TPS’s optimizer, and no further adjustments are made during optimization. Unlike KBP, AP relies on the optimization algorithm itself to iteratively adjust planners’ pre-set, PTVs/OARs DVH objectives during optimization to meet or exceed coverage/sparing goals. In APMCO, planners are free to use the valuable knowledge from AP and to adjust quickly and efficiently the allowable minimum dose to the OARs with confidence that the dose to the target and other tissues will remain uncompromised, thus greatly reducing the time-consuming iteration loops.

A wide range of mathematically optimal alternatives are available during navigation. As a result, it suggests a paradigm shift in the decision making process during which the physician gets an insight of the solution space by navigating through alternative compromises. Voet et al. [38] used the prioritized optimization, resulting in one treatment plan only, and not requiring manual Pareto surface navigation. The in-house-developed algorithm is promising but not yet commercially available. Just as our experience in using MCO, navigating to the clinically optimal plan from the Pareto surface plan database is equally critical to achieving high quality plans. It involves higher-level information which is often non-technical, qualitative and experience-driven. The OARs sparing in MCO plans also reflects the ability of physicians to prioritize a lot of different structures during the final plan selection in the navigation step. For example, the routine clinical practice is that under-dosing to PTVs and critical OARs placed in high priority level will be focused on and unspecified tissues tend to be less important. Appreciable variation exists in the navigation step, depending on the experience of individual physicians. Due to lacking in the knowledge of such a wide variety of tradeoff solutions, the decision making for how to cope with the problem is rather demanding especially for physicians who have few experiences during navigation. Although physicians can choose a scalarized objective and find the resulting solution, there are no guarantees that they can derive the optimal solution every time even when experienced ones are involved. However, once the individual and accurate tradeoff information is obtained from AP, no matter whether physicians are experienced or not can ensure navigation in a more efficient and straightforward manner.

The Pareto surface plan database is very large-dimensional since NPC has multiple PTVs and many individual OARs to consider. Reducing the dimensionality of the database is a very important consideration. Kierkels et al. [39] demonstrated that the application of clinically validated multivariable normal tissue complication probability (NTCP) models (including multiple dose parameters and prognostic clinical factors) in the planning process facilitated the dose distributions and corresponding NTCP-estimates in head and neck cancer patients. It may be more appropriate, especially in a multicriteria setting, to reduce the number of currently used tradeoff objectives to a few NTCP-based objectives, simplifying the navigation process. This is a promising area and definitely more computationally faster objective-reduction techniques are needed for the purpose.

One important limitation for MCO is that the Pareto surface generation step is computationally costly. Clinical constraints of time and effort also should be kept a close eye on. To our knowledge, the time expense of computing a modest number of fluence-based plans can exceed 5 h for complicated NPC cases. Furthermore, APMCO VMAT required additional average of 5 h compared to general MCO VMAT. The search for AP plans is not in a time-efficient manner since the optimizer will run multiple times with adjustments of optimization parameters. This situation becomes especially challenging in triple-arc VMAT plans. The clinically infeasible planning time seems to severely limit the usability. Special caution should be taken when APMCO is applied in the clinical practice. Fortunately, most of the planning time is not “active planning” time which does not require active manual intervention. Improvements in computer architecture, e.g. by calculation on the graphics processing unit, or generation of plans in parallel over multiple workstations might drastically reduce the planning time. For early-stage NPC cases, VMAT plans generated using only one arc may greatly reduce the planning time and seem to be well tolerable. Another limitation is that AP optimization and MCO are entirely two separate processes. An ideal procedure is to incorporate simultaneously AP into the MCO framework. APMCO should be more usable in practice in the future. The last limitation is that we just focused on recommending a new strategy for MCO in this study. Further studies are therefore needed to go into the mathematical details of the optimization algorithm. In principal, the new optimization approach can also be used for other site tumors such as thorax, abdomen, etc.

## Conclusion

A new strategy for making the appropriate choice of the representative optimization parameters in planning processes and accurate selection criteria during Pareto surface navigation for general MCO was recommended. The APMCO approach combines both benefits of AP optimization and MCO for achieving an individual VMAT plan according to the patient-specific tradeoff among conflicting priorities at a cost of increased planning time which does not require active manual intervention. The potential of the APMCO strategy is best realized with a clinical implementation that exploits individual generation of Pareto surface representations without manual interaction. It also assists physicians to ensure navigation in a more efficient and straightforward manner.

## Abbreviations

AP: AutoPlanning
APMCO: AP optimization and MCO
CN: Conformation number
CTV: Clinical target volumes
D_2%_: Near maximum dose
D_98%_: Near minimum dose
D_mean_: Mean dose
DVH: Dose-volume histogram
KBP: Knowledge-based planning
MCO: Multicriteria optimization
MU: Monitor unit
NPC: Nasopharyngeal carcinoma
NTCP: Normal tissue complication probability
OAR: Organ at risk
PTV: Planning target volume
RTOG: Radiation Therapy Oncology Group
TPS: Treatment planning system
V_30_: The percent of volume that received 30Gy
VMAT: Volumetric-modulated arc therapy
δ: The relative dose deduction
